# CDK2AP1 influences immune infiltrates and serves as a prognostic indicator for hepatocellular carcinoma

**DOI:** 10.3389/fgene.2022.937310

**Published:** 2022-08-29

**Authors:** Yibin Che, Ge Wang, Qiang Xia

**Affiliations:** ^1^ Department of Liver Surgery, Renji Hospital, School of Medicine, Shanghai Jiao Tong University, Shanghai, China; ^2^ State Key Laboratory of Oncogenes and Related Genes, Renji-Med X Clinical Stem Cell Research Center, Ren Ji Hospital, Shanghai Cancer Institute, School of Medicine, Shanghai Jiao Tong University, Shanghai, China; ^3^ Shanghai Engineering Research Center of Transplantation and Immunology, Shanghai, China; ^4^ Shanghai Institute of Transplantation, Shanghai, China

**Keywords:** CDK2AP1, hepatocellular carcinoma, immune infiltrate, prognosis, biomarker

## Abstract

**Background:** Hepatocellular carcinoma (HCC) is a tumor with high malignancy and poor 5-years survival rate. Excellent tumor markers are very important for early clinical diagnosis and prognosis evaluation. Previous studies have shown that *CDK2AP1* (Cyclin-dependent kinase 2-associated protein 1) is involved in cell-cycle and epigenetic regulation. In the present study, we assess *CDK2AP1* expression, prognostic value, immunomodulatory and possible influencing pathways in HCC.

**Method:** The Cancer Genome Atlas (TCGA) database was used to analyse gene expression, clinicopathology and prognosis. The protein level of CDK2AP1 in HCC tissues was detected in the Human Protein Atlas (HPA) database. The immune score in HCC to *CDKAP1* expression were analyzed using ESTIMATE. Furthermore, we use Tumor IMmune Estimation Resource (TIMER) database to study *CDK2AP1* expression and Immune Infiltration Levels in HCC. Co-expressed genes of *CDK2AP1* were predicted and elaborated by LinkedOmics.

**Results:** In normal liver tissues, the expression of *CDK2AP1* was significantly lower than tumor tissues, and was correlated with the level of clinical stage and histologic grade in HCC patients. Patients with high expression of *CDK2AP1* have a poor prognosis than patients with low *CDK2AP1* expression. *CDK2AP1* expression level exhibits significantly positive correlations with the number of infiltrating B cells, CD4^+^ T cells, CD8^+^ T cells, Macrophages, Neutrophils, and DCs in HCC tissues. KEGG enrichment analysis showed that the related pathways affected by *CDK2AP1* mainly include: Fc gamma R-mediated phagocytosis, Th1 and Th2 cell differentiation, Cell cycle, etc. Both *in vitro* and *in vivo* experiments confirmed that *CDK2AP1* promotes the proliferation and metastasis in hepatocellular carcinoma. Our results highlight the role of *CDK2AP1* as an important prognostic indicator and immunotherapy target for HCC patients.

**Conclusion:** We found *CDK2AP1* as a new prognostic biomarker for HCC, which could help explain changes in the biological processes and immune environment lead to liver cancer development. Therefore, *CDK2AP1* is a potential new target for HCC therapy.

## Introduction

Hepatocellular carcinoma accounts for about 75–85% of liver cancers (Bray et al., 2018). It is a highly malignant tumor (Yang et al., 2019). Due to the lack of specific biomarkers for early diagnosis and the inconspicuous symptoms, the patients are often at an advanced stage when discovered (Liu et al., 2019). It currently ranks fourth in the world in cancer-related mortality and therefore has a greater impact on human health (Villanueva, 2019). Although many studies have been carried out, there are still few biomarkers with high specificity and high sensitivity for early detection and prognosis evaluation of HCC (Zhang and Wang, 2021). In addition to traditional chemotherapy drugs, immunotherapy also plays an important role in the treatment of HCC. Immune checkpoint inhibitors PD1 antibodies (nivolumab and pembrolizumab) and PD-L1 antibodies (atezolizumab) have been used clinically, but immunotherapy is only effective in a small number of patients (Finn et al., 2020; Yau et al., 2020; Yau et al., 2022). Effective immunotherapy strategies for HCC will require a deeper understanding of key changes in the tumor microenvironment.

CDK2AP1 is a cyclin-dependent kinase 2 (CDK2)-related protein that negatively regulates CDK2 activity by proteolytically hydrolyzing CDK2 (Matsuo et al., 2000). Various evidences suggest that *CDK2AP1* modulates the actions of transforming growth factor-B1 (TGFB1) and retinoblastoma (Rb) protein to suppress tumor growth (Hu et al., 2004; Zolochevska and Figueiredo, 2010). The protein also interacts with DNA polymerase alpha to regulate DNA replication in the S phase of the cell cycle (Tadesse et al., 2019). In head and neck cancer, *CDK2AP1* inhibits CDK2/CyclinE activity, while under normal conditions CDK2/CyclinE can phosphorylate Retinoblastoma Protein (Rb), release E2F, and then transcribe the required components of the cell, subsequently going through the G1/S phase transition (Figueiredo et al., 2005). In human oral squamous cell carcinoma (OSCC), decreased expression of TGF-β receptor II (TGFβRII) and *CDK2AP1* leads to cell proliferation and survival (Prime et al., 2004; Peng et al., 2006). However, to the best of our knowledge, the expression and mechanism of *CDK2AP1* in hepatocellular carcinoma have not been reported yet. Considering that *CDK2AP1* may be a potential target for the diagnosis and treatment of HCC, we conducted the following studies.

First, we checked the expression of *CDK2AP1* at the transcriptional level and protein level in the TCGA database and the HPA database, respectively. We observed that the expression level in tumors was higher than normal tissues. Survival analysis and ROC curves were used to show the prognostic value of *CDK2AP1*. Univariate Cox, multivariate Cox, and prognostic models were established to explore the role of *CDK2AP1* as an independent risk factor for prognosis in patient risk assessment.

Afterwards, we discussed the relationship between *CDK2AP1* and neoantigens, microsatellite instability (MSI), tumor mutation burden (TMB), and immune infiltration, which is helpful to further explore the immunotherapy of HCC (Liu et al., 2021; Zeng et al., 2021). Previous studies have demonstrated that tumor neoantigens can enhance T cells reactivity and guide the personalized treatment of patients (Schumacher and Schreiber, 2015; Ott et al., 2017). TMB is generally considered to be associated with immune infiltration and has implications in predicting response to immunotherapy and overall survival (Wong et al., 2021). Kyoto Encyclopedia of Genes and Genomes (KEGG) and Gene ontology (GO) pathway enrichment analysis further predicted the potential biological pathway of *CDK2AP1*. In conclusion, these results provided insights for new therapeutic strategies for hepatocellular carcinoma.

## Materials and methods

### Data collection and analysis

We obtained the expression data of *CDK2AP1* in pan-cancer in The Cancer Genome Atlas (TCGA) database, and extracted the clinical information of LIHC for subsequent analysis. HCC patients from the ICGC LIVER CANCER - RIKEN, JP cohort were used for model validation. Subsequent analysis was implemented by R version 4.0.3. *CDK2AP1* expression in tumor cells and normal cells were explored with BioGPS database (Wu et al., 2016). The Human Protein Atlas Database (HPA) (www.proteinatlas.org) has immunohistochemical images of a variety of tumors, which were used to analyze CDK2AP1 protein expression between normal and hepatocellular carcinoma tissues. Neoantigen profiles were analyzed for a variety of cancer types from the Cancer Genome Atlas (TCGA) cohort. Neoantigen, TMB and MSI were analyzed in different cancers using the SangerBox (http://www.sangerbox.com/tool), a free online platform for data analysis. The correlation between *CDK2AP1* expression and the immune or molecular subtypes of HCC was achieved through TISIDB database (Ru et al., 2019).

### Linkedomics database

The LinkedOmics database includes multiple omics data and clinical data for 32 cancer types, and it also includes mass spectrometry-based proteomics data for a variety of tumors (Vasaikar et al., 2018). The LinkedOmics database was used for co-expressed gene analysis. The top 50 genes positively and negatively associated with *CDK2AP1* in LIHC were obtained through the LinkFinder module. KEGG and GO enrichment analysis of co-expressed genes were carried out in LinkInterpreter module.

### Analysis of tumor-infiltrating immune cells

ESTIMATE algorithm was used to generate ImmuneScore, StromalScore, and EstimateScore for each HCC patient in the Cancer Genome Atlas. The correlation between *CDK2AP1* expression and tumor immune infiltration (B cells, CD4^+^ T cells, CD8^+^ T cells, Macrophages, Neutrophils, and DCs) was explored via Tumor IMmune Estimation Resource (TIMER).

### Cell transfection

The shRNA targeting the protein-coding region of the human CDK2AP1 gene (NCBI gene ID: 8,099) was designed and synthesized as follows: shRNA1, 5′- CAT​GGC​AAC​GTC​TTC​ACA​GTA-3'; shRNA2, 5′- CAT​GGC​AAC​GTC​TTC​ACA​GTA-3'. The shRNA targeting the protein-coding region of the mouse CDK2AP1 gene (NCBI gene ID: 13445) was designed and synthesized as follows: shRNA, 5′-GCT​TGG​CTG​AAA​CGG​AAC​GGA-3'. HCCLM3, MHCC97H and Hepa1-6 cells in logarithmic growth phase were transfected with LipofectamineTM 2000. The results were verified by Western Blotting after screening with puromycin at 5 μg/ml.

### Western blotting assay

Total protein was isolated from cells using RIPA lysis buffer. Protein concentration was determined using the BCA protein quantification kit, after which equal amounts of protein samples were loaded on SDS-PAGE, transferred to PVDF membranes, and immunoblotted with primary antibodies against CDK2AP1 (Abcam) and GAPDH (Abcam) and peroxidase-labeled secondary antibodies (CST). ECL Reagent (Bio Rad) was used to visualize immune response bands.

### CTG assay

Cell proliferative capacity was detected using the Cell Titer-Glo Cell Viability Kit (Promega, Madison, WI). The cells were seeded into 96-well plates at 4,000 cells/well, and the proliferation of the two groups of cells was measured at different time points. Add 50 μl of detection reagent to each well and detect with GloMax^®^ luminometer.

### Cell migration and invasion assay

Cell migration and invasion assays were used to assess cell migration and invasion capabilities. For migration experiments, cells were resuspended in 200 μl of serum-free DMEM and plated in the upper chamber of the transwell at a density of 5 × 10^4^ cells per well. 600 μl of DMEM containing 10% FBS was added to the lower chamber to attract cells. Then, incubate in a 37°C incubator for 24 h. For invasion experiments, 5 × 10^4^ cells were plated in the upper chamber, which had been filled with 40 μl Matrigel. After 24 h of culture, the number of migrated or invaded cells was counted under light microscopy after fixation with 4% paraformaldehyde and staining with 0.1% crystal violet.

### Animal experiment

6–8 week old C57/BL6 mice were used for the construction of orthotopic tumor models. 10^6^ Hepa1-6 or CDK2AP1 knockdown Hepa1-6 cells were injected into the liver lobes of mice, mice were sacrificed 2 weeks after tumor seeding, and liver tumor diameters were recorded. In addition, we recorded the survival of mice every day for 6 weeks after tumor implantation and plotted survival curves. All mice were housed in specific pathogen-free (SPF) conditions with access to sterile water and food. All animal procedures were approved by the Institutional Animal Care and Use Committee of the Shanghai Jiao Tong University School of Medicine.

### Statistical analysis

The RNA sequencing expression profile and corresponding clinical information of LIHC were downloaded from the TCGA dataset (https://portal.gdc.com). According to the expression of *CDK2AP1*, patients in the LIHC cohort were divided into two groups with high and low expression, and the overall survival rates of the two groups were compared using Kaplan-Meier survival analysis. Univariate and multivariate Cox regression analyses were conducted to evaluate the relationship of *CDK2AP1* expression and other clinicopathological factors (age, sex, TNM stage, and grade) on survival. The forest is used to display the *p* value, HR, and 95% CI for each variable. Based on a multivariate Cox proportional risk analysis, a nomogram was developed for predicting overall recurrence at 1, 2, 3 and 5 years. The nomogram provides a graphical representation of factors that can be used to calculate an individual patient’s risk of recurrence by points associated with each risk factor. The associations between variables were analyzed by means of Student’s t tests, Mann-Whitney U tests as appropriate. All *p* < 0.05 results were considered statistically significant.

## Results

### The differential expression of CDK2AP1 in LIHC

**FIGURE 1:**
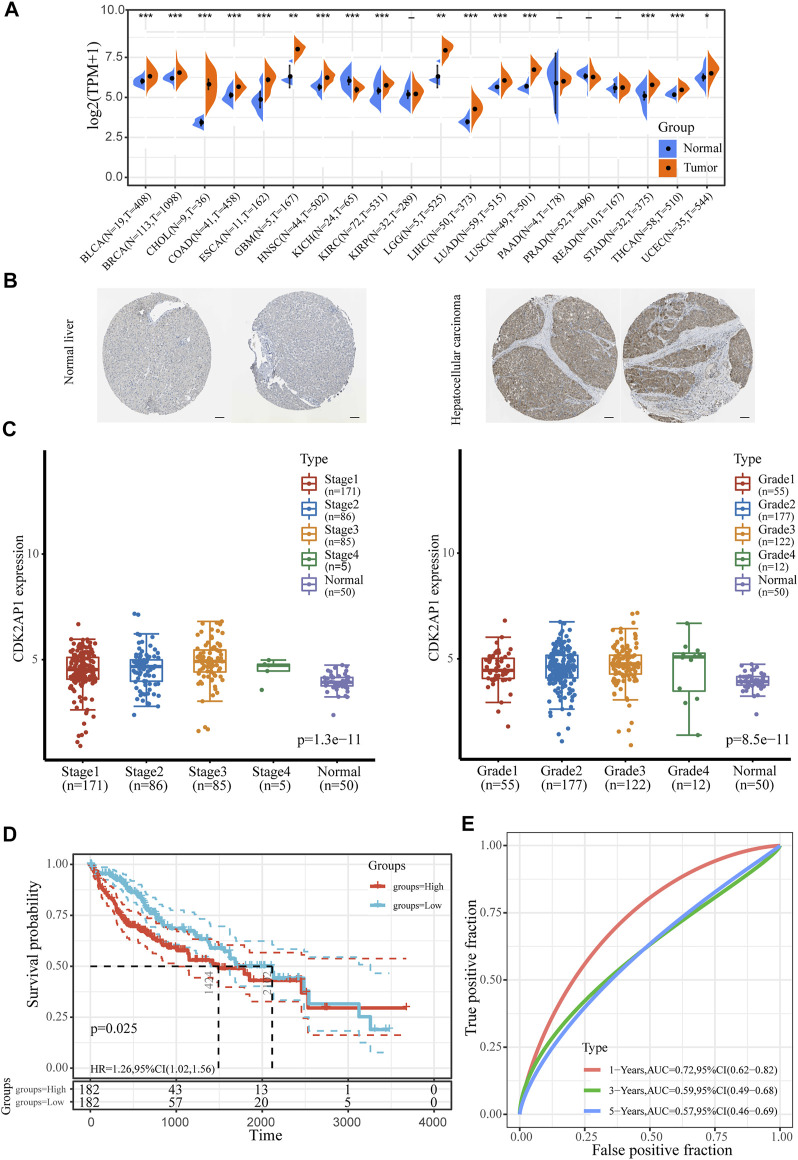
The expression level of *CDK2AP1* in LIHC **(A)** Pan-cancer analysis of the expression of *CDK2AP1* **(B)** The expression of *CDK2AP1* protein in normal liver and hepatocellular carcinoma tissues was observed by immunohistochemistry using HPA database. Scale bars, 100 μm **(C)** *CDK2AP1* expression was significantly correlated with clinical stage and histologic grades **(D)** Patients with high expression of *CDK2AP1* had inferior OS than those with low expression of *CDK2AP1* **(E)** ROC curves of *CDK2AP1*. *p < 0.05, **p < 0.01 and ***p < 0.001.

Pan-cancer sequencing data obtained from TCGA database were used to compare *CDK2AP1* expression in 20 different types of tumor and corresponding normal tissues. The results showed the level of *CDK2AP1* expression is up-regulated in breast invasive carcinoma (BRCA), bladder urothelial carcinoma (BLCA), colon adenocarcinoma (COAD), cholangio carcinoma (CHOL), liver hepatocellular carcinoma (LIHC), Esophageal carcinoma (ESCA), Glioblastoma multiforme (GBM), head and neck squamous cell carcinoma (HNSC) and eight other tumors while down-regulated in Kidney Chromophobe (KICH) (Figure 1A). BioGPS database was used to show the expression levels of *CDK2AP1* in 10 cancer cell lines and 10 normal cell lines. The expression level of *CDK2AP1* in normal cells was significantly lower than that in tumor cells (Supplementary Figures S1A,B). Immunohistochemical results obtained from the Human Protein Atlas (HPA) database showed that *CDK2AP1* was expressed at a higher level in HCC tumor tissues than in non-tumor tissues (Figure 1B). Moreover, by comparing the expression of *CDK2AP1* in LIHC samples at different clinical stages and histologic grades, we found that increased expression of *CDK2AP1* associated significantly with clinical stage (*p* < 0.001) and tumor grade (*p* < 0.001, Figure 1C). The distribution of *CDK2AP1* expression and the survival status of HCC patients were shown in Supplementary Figure S1C. HCC patients with higher risk score had poorer overall survival rate and higher mortality. Besides, an analysis of the Kaplan–Meier survival data indicated high expression level of *CDK2AP1* was associated with a poor prognosis of OS (median OS 1424 vs. 2,102 days, *p* = 0.025, Figure 1D). The expression of *CDK2AP1* showed excellent prognostic ability, because ROC curve showed that the AUC of *CDK2AP1* expression predicting survival at 1, 3 and 5 years was 0.72, 0.59 and 0.57 (Figure 1E).

### Construction of LIHC prognostic model

**FIGURE 2:**
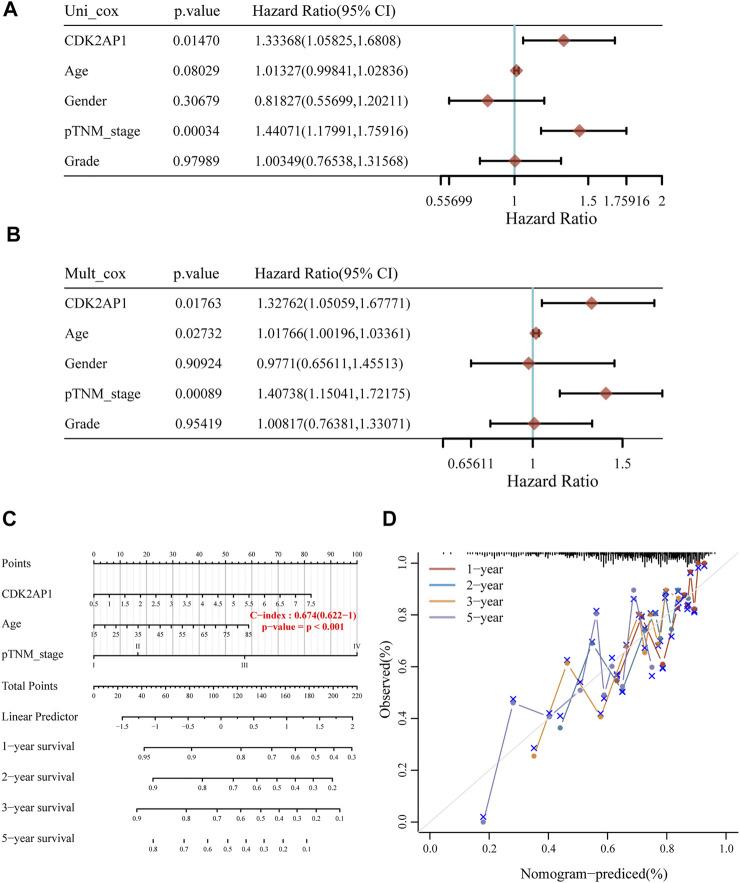
Construction of LIHC prognostic model **(A)** Univariate Cox analysis of the risk factors related with survival of LIHC **(B)** Multivariate Cox regression analysis of the risk factors related with survival of LIHC **(C)** The nomogram model can predict the prognosis of HCC patients **(D)** The calibration curves of the overall survival nomogram model. An ideal nomogram is represented by the dashed diagonal line, while the 1-year, 2-years, 3-years and 5-years observed nomograms are illustrated by the red, blue, orange and purple lines.

In order to determine the risk factors related with LIHC survival, we used both univariate and multivariate Cox regression analyses. Univariate Cox analysis indicated that TNM stage (*p* < 0.001) and expression of *CDK2AP1* (*p* = 0.0147) were associated with overall survival (Figure 2A). We further conducted multivariate Cox regression analysis, depicted as a forest boxplot in Figure 2B, which showed that age (*p* = 0.027), TNM stage (*p* < 0.001) and expression of *CDK2AP1* (*p* = 0.017) were independent predictors of LIHC prognosis. To calculate the patient’s prognostic risk, we established a nomogram containing the above risk factors (Figure 2C). Nomogram’s C-index is 0.674. Calibration curves for 1 year, 2, 3, and 5 years of survival in the discovery cohort indicate good consistency between prediction and observation (Figure 2D). We used the LIVER CANCER - RIKEN, JP data from ICGC database as the external validation of the nomogram. The C-index of the prediction model in the validation cohort is 0.649, and the discrimination and calibration curves indicated that the prediction model also has good performance in the validation set (Supplementary Figure S2).

### Immunological aspects related to CDK2AP1 in LIHC

**FIGURE 3:**
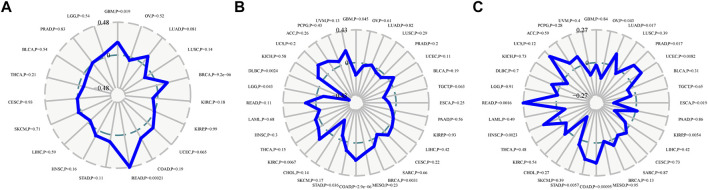
Immune score and the expression of *CDK2AP1* in LIHC **(A)** Correlation analysis radar diagram of *CDK2AP1* expression and neoantigen **(B)** Correlation analysis radar diagram of *CDK2AP1* expression and MSI **(C)** Correlation analysis radar diagram of *CDK2AP1* expression and TMB.

**FIGURE 4:**
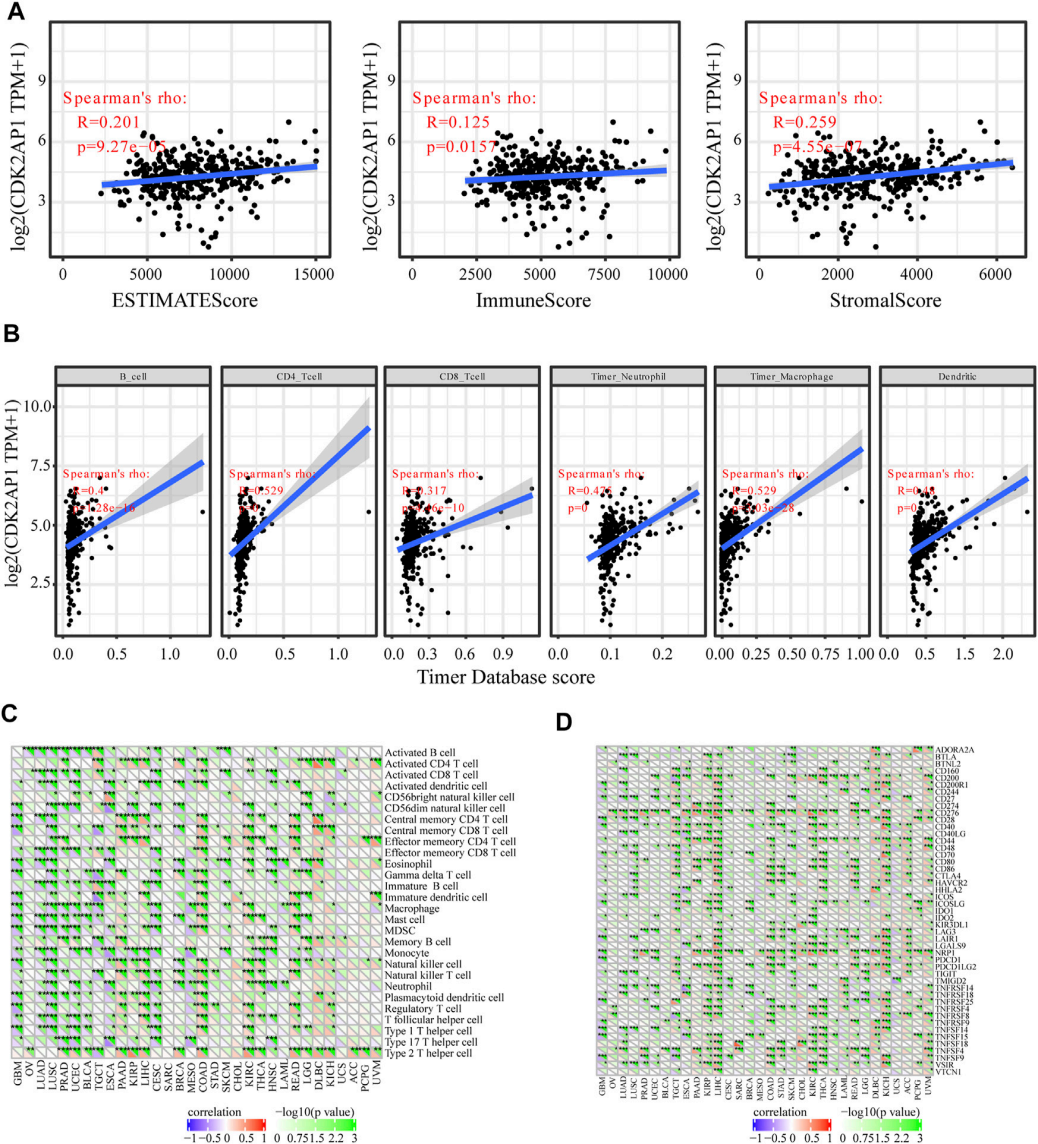
Immunological aspects related to *CDK2AP1* in LIHC **(A)** The correlation analysis of *CDK2AP1* expression and infiltrating stromal/immune cells in tumor tissues using ESTIMATE algorithm **(B)** the correlation analysis of *CDK2AP1* expression and composition of infiltrating immune cells in tumor samples **(C)** The correlation analysis of *CDK2AP1* expression and acknowledged markers of immune pathway **(D)** The correlation analysis of *CDK2AP1* expression and immune checkpoint genes. *p < 0.05, **p < 0.01 and ***p < 0.001.

Neoantigens, microsatellite instability and tumour mutation burden can be used as tumor immunotherapy targets and were closely related to the efficacy of tumor immunotherapy, thus providing the possibility for the realization of precise immunotherapy (Westcott et al., 2021; Ma et al., 2022). The correlation analysis of *CDK2AP1* with neoantigens, TMB and MSI showed that it had less effect on these biomarkers in HCC, with *p* values of 0.59, 0.42 and 0.42, respectively. The correlation between the expression level of *CDK2AP1* and neoantigens was performed, which indicated that *CDK2AP1* expression was significant related to neoantigens in GBM (*p* = 0.019), BRCA (*p* = 9.2e-06) and rectum adenocarcinoma (READ) (*p* = 0.00021) (Figure 3A). Furthermore, *CDK2AP1* was also found correlated with TMB in GBM (*p* = 0.045), BRCA (*p* = 0.0031), COAD (*p* = 2.9e-06), Stomach adenocarcinoma (STAD) (*p* = 0.036), Kidney renal clear cell carcinoma (KIRC) (*p* = 0.0067), Brain Lower Grade Glioma (LGG) (*p* = 0.043) and Lymphoid Neoplasm Diffuse Large B-cell Lymphoma (DLBC) (*p* = 0.0024) (Figure 3B) and correlated with MSI in Ovarian serous cystadenocarcinoma (OV) (*p* = 0.043), Lung adenocarcinoma (LUAD) (*p* = 0.017), Prostate adenocarcinoma (PRAD) (*p* = 0.017), Uterine Corpus Endometrial Carcinoma (UCEC) (*p* = 0.0082), ESCA (*p* = 0.019), Kidney renal papillary cell carcinoma (KIRP) (*p* = 0.0054), COAD (*p* = 9.5e-04), STAD (*p* = 0.0057), HNSC (*p* = 0.0023), READ (*p* = 0.0016) (Figure 3C). In addition, we used TIMER and ESTIMATE to explore the association between *CDK2AP1* expression and tumor microenvironment related immune cells, stromal cells, and tumor cells (Figure 4A). The ESTIMATE data indicated no statistically significant link between *CDK2AP1* expression and the tumor microenvironment. Using the TIMER tool, we also assessed the possible association between *CDK2AP1* expression and the level of HCC immune infiltration. As shown in Figure 4B, *CDK2AP1* expression levels were significantly correlated with B cells, CD4^+^ T cells, CD8^+^ T cells, Neutrophils, Macrophages, and DCs. Our results concluded that *CDK2AP1* expression level is associated with poor prognosis and high immune infiltration in HCC. Afterwards, the role of *CDK2AP1* expression in HCC immune and molecular subtypes was explored through the TISIDB database. The results showed that the expression of *CDK2AP1* in HCC is related to different immune and molecular subtypes, specifically in C1 immune subtype and iCluster1 molecular subtype with higher *CDK2AP1* expression (Supplementary Figures S1D,E). To further identify potential targets for LIHC tumor immunotherapy, we used RNAseq data from the TCGA database to check the relationship between *CDK2AP1* expression and immune-related pathways and checkpoint genes. Analysis of *CDK2AP1* expression and immune pathway reveals that *CDK2AP1* affects the immune pathway of activated B cell, activated CD4 T cell, activated dendritic cell, central memory CD4 T cell, central memory CD8 T cell, effector memeory CD4 T cell, natural killer cell and ten other types of immune cells (Figure 4C). Based on the correlation analysis of *CDK2AP1* and the checkpoint genes, *CDK2AP1* expression was correlated with almost all checkpoint genes except *ADORA2A*, *BTNL2*, *ID O 2*, *TNFRSF9* and *TNFSF14* (Figure 4D). Therefore, we could propose that *CDK2AP1* may influence the tumor immune microenvironment so that patients with high *CDK2AP1* levels probably benefit more from immunotherapy.

### CDK2AP1 co-expression network and enrichment analysis of genes

**FIGURE 5:**
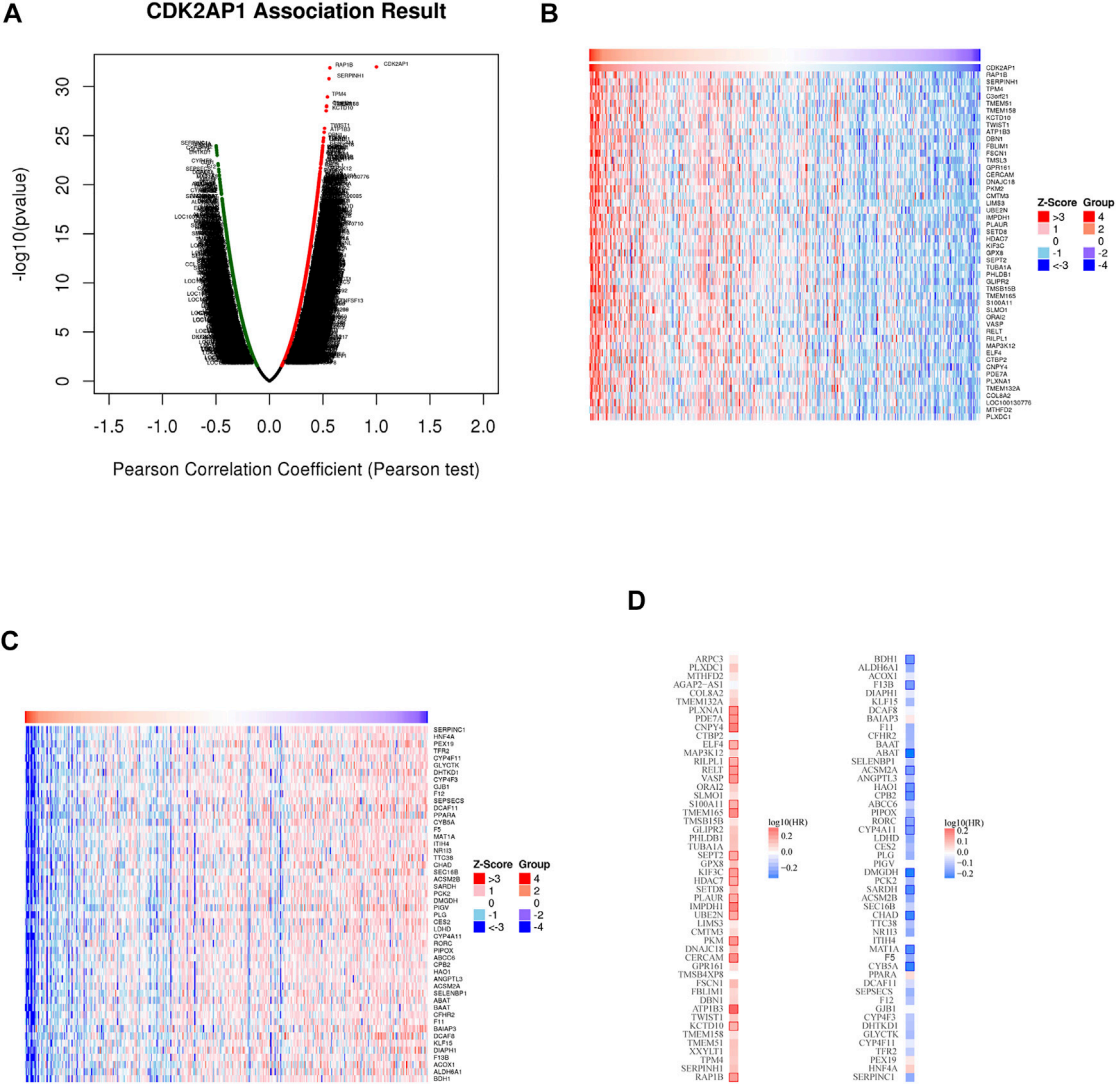
The co-expression genes with *CDK2AP1* in LIHC **(A)** Genes significantly associated with *CDK2AP1* were distinguished by Pearson test in LIHC cohort. Red indicates positively related genes and green indicates negatively related genes **(B,C)** Heatmaps show the top 50 genes positively and negatively related to *CDK2AP1* in LIHC **(D)** Gene survival map of the top 50 genes that are positively and negatively associated with *CDK2AP1* in LIHC.

To further illustrate the role of *CDK2AP1* in tumor biological process, we used the Linkedomics database to explore the co-expression pattern of *CDK2AP1* (Figure 5A). According to the *p* value and Pearson correlation coefficient, the top 50 genes with positive and negative correlation were determined (Supplementary Tables S1, S2). The heatmap shows the relationship between *CDK2AP1* expression and positive and negative related genes (Figures 5B,C). Next, we compared the survival contribution of positive and negative related genes in LIHC cohort (Figure 5D). What caught our attention was that the proportion of the top 50 positively correlated genes as high-risk markers in LIHC was very high, on the contrary, a large proportion of the top 50 negatively correlated genes (42/50) had protective effects on HCC. This is consistent with the previous results of *CDK2AP1*.

**FIGURE 6:**
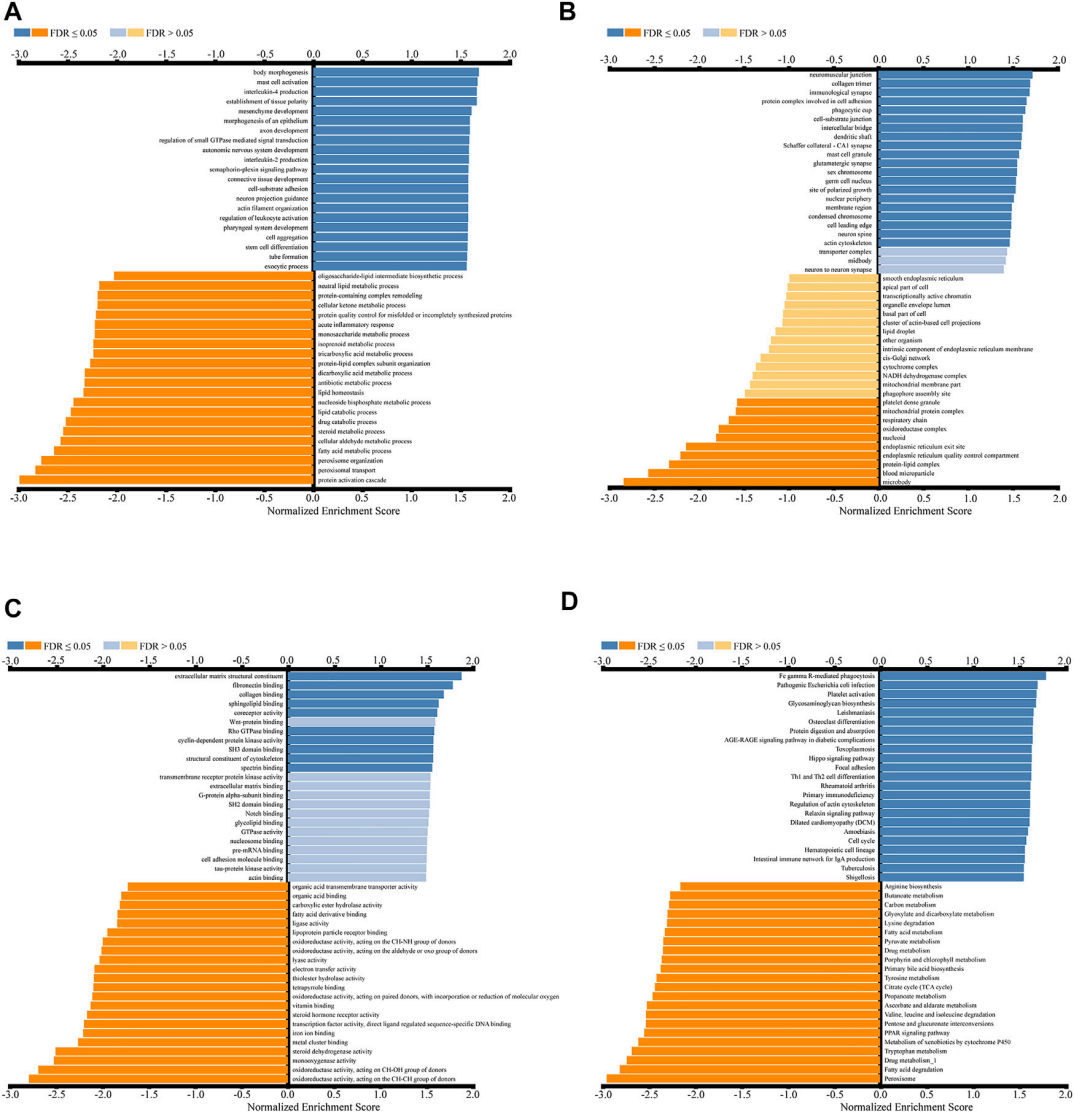
Enrichment analysis results of *CDK2AP1*-related genes in LIHC cohort **(A–C)** GO gene enrichment analysis diagram of *CDK2AP1*-related genes with biological processes **(A)**, cellular components **(B)** and molecular functions **(C)** categories **(D)** KEGG enrichment analysis diagram of *CDK2AP1*-related genes.

GO analysis showed that *CDK2AP1* co-expressed genes in biological process (BP) classification mainly focused on mast cell activation, interleukin-4 production, mesenchyme development, Regulation of leukocyte activation and stem cell differentiation (Figure 6A). Cellular Component (CC) classification mainly focuses on immunological synapse, protein complex involved in cell adhesion, Dendritic shaft and Actin cytoskeleton (Figure 6B). In the category of molecular function (MF), these genes were enriched in extracellular matrix structural constituent, fibronectin binding, Wnt-protein binding, cyclin-dependent protein kinase activity and G-protein alpha-subunit binding (Figure 6C).

KEGG enrichment analysis indicated that co-expressed genes were mainly enriched in Fc gamma-r-mediated phagocytosis, Glycosaminoglycan biosynthesis, Protein Digestion and absorption, Hippo Signaling Pathway, Th1 and Th2 cell differentiation, and Intestinal immune network for IgA production, etc (Figure 6D, Supplementary Table S3).

### CDK2AP1 inhibits tumor progression *in vitro* and *in vivo*

**FIGURE 7:**
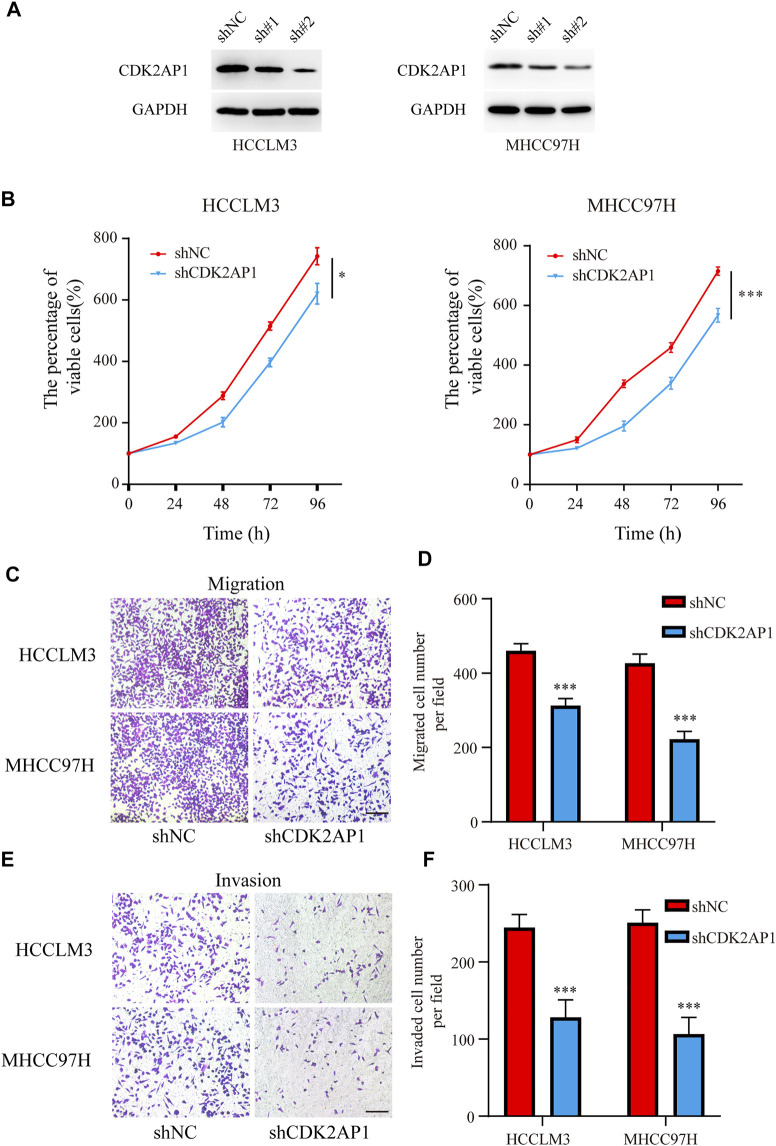
CDK2AP1 promotes proliferation and migration of HCC cell lines *in vitro* **(A)** Western blotting of CDK2AP1 in HCCLM3 and MHCC97H cells transfected with CDK2AP1-shRNA **(B)** Proliferative curve of HCCLM3 and MHCC97H cells at indicated times **(C)** The migration assay of HCCLM3 and MHCC97H cells in two groups **(D)** Quantification of the migration ability **(E)** The invasion assay of HCCLM3 and MHCC97H cells in two groups **(F)** Quantification of the invasion ability. *p < 0.05, **p < 0.01 and ***p < 0.001.

**FIGURE 8:**
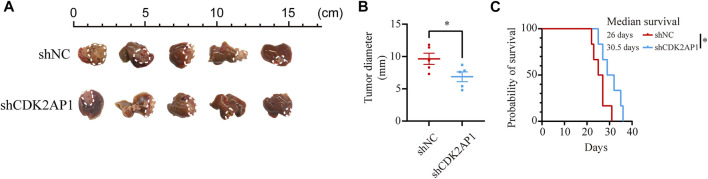
CDK2AP1 promotes tumor growth and decreases overall survival *in vivo* **(A)** Photographs of tumors in orthotopic tumor models **(B)** Tumor diameter of two groups was measured at the end of the experiment **(C)** The mice survival curves after tumor inoculation. *p < 0.05, **p < 0.01 and ***p < 0.001.

To verify the role of *CDK2AP1* in HCC development, we performed experiments *in vitro* and *in vivo*, respectively. First, we constructed *CDK2AP1* knockdown HCCLM3 and MHCC97H cells (Figure 7A). Cell proliferation experiments showed that after knockdown of *CDK2AP1*, the proliferation ability of HCC cells was significantly inhibited (Figure 7B). Cell migration and invasion experiments also demonstrated that knockdown of *CDK2AP1* significantly inhibited the migration and invasion abilities of HCC cells (Figures 7C–F). Subsequent *in vivo* experiments showed that the diameter of liver tumors in the *CDK2AP1* knockdown group was significantly smaller than that of the control group (Figures 8A,B), and the median survival time of the two groups was 30.5 and 26 days (Figure 8C), respectively.

## Discussion

HCC is one of the most common tumors worldwide, making it the fourth leading cause of cancer-related mortality worldwide (Dai et al., 2021). Its occurrence is often accompanied by related gene mutations, molecular signaling pathways and epigenetic changes (El-Serag and Rudolph, 2007; D'Souza et al., 2020). Surgical treatment methods mainly include liver tumor resection, transarterial chemoembolization (TACE), ablation and liver transplantation, and drug treatment includes small molecule targeted drugs and monoclonal antibodies, but the overall clinical treatment effect is not satisfactory (Raoul et al., 2019; Llovet et al., 2021). With the development of immune therapy, immunotherapy of HCC has played an increasingly important role (Kang et al., 2022). The previous study found tumor progression in patients with HCC is related to immune pathways. Tumor tissues have more lymphocyte infiltration and inflammation in the related pathways often herald better immune therapy effect, as well as the overall prognosis (Zheng et al., 2021). The prediction of immunotherapy efficacy mainly depends on PD-L1 expression, TMB, microsatellite instability (MSI)/mismatch gene repair (MRR) status, tumor-infiltrating lymphocytes (TIL) and Treg cells, etc (Zekri et al., 2005). However, the proportion of MSI-H in liver cancer is very low, and some other biomarkers still fail to achieve the expected clinical effect (Wang et al., 2001). In view of this, efforts should be made to explore more biomarkers and develop more immunotherapy target drugs. This study aims to explore the biological mechanism of HCC and find meaningful molecular markers or therapeutic targets in order to find new diagnostic methods and safe therapeutic approaches for HCC.

Cell cycle is a basic biological process that maintains cell life activities, and it is mainly regulated by Cyclins, cyclin-dependent protein kinases (CDKs) and cyclin-dependent protein kinase inhibitors (CKIs) (Malumbres and Barbacid, 2009; Bertoli et al., 2013). They regulate each other to achieve precise regulation of cell cycle. The malignant proliferation of tumor cells is due to the imbalance between growth-promoting and growth-inhibiting signals (Gerdes, 2002). *CDK2AP1* is a cyclin-dependent kinase 2 (CDK2)-related protein and is involved in the regulation of DNA replication during the S phase of the cell cycle (Wong et al., 2012). Previous studies have demonstrated that *CDK2AP1* influence tumor growth such as glioma, lung cancer and esophageal squamous cell carcinoma (Hiyoshi et al., 2009; Sun et al., 2013; Xu et al., 2014). Knockdown of *CDK2AP1* significantly inhibits glioma cell proliferation and induces cell cycle arrest in G0/G1 phase, thus speculating that *CDK2AP1* may be an anti-apoptotic molecular switch in glioma development. In lung cancer, however, the antitumor activity of *CDK2AP1* may act through affecting cell cycle regulators other than CDK2. The researchers also found that *CDK2AP1* overexpression enhanced chemosensitivity to cisplatin and paclitaxel (Sun et al., 2013). Controversial results of *CDK2AP1* in different tumor types suggest a complex regulatory mechanism.

Through bioinformatics mining of public databases, this study elaborated the role of *CDK2AP1* in the immune microenvironment and the cellular biological pathways involved in HCC. Considering the potential function of *CDK2AP1*, we attempted to explore the possibility of *CDK2AP1* as a new target of action.

By performing differential expression analysis on HCC patients in the TCGA database, we found that *CDK2AP1* was significantly downregulated in HCC samples compared with normal tissues. Likewise, by comparing samples of different stages and grades, we noticed that *CDK2AP1* was upregulated in relatively high malignant or advanced stages of HCC. These findings demonstrate the important role of *CDK2AP1* in the occurrence and development of HCC. To clarify the important role of *CDK2AP1* in the prognostic evaluation of HCC, we performed univariate Cox, multivariate Cox analysis, and survival analysis using patient data from the LIHC cohort. The results suggest that patients with high expression have a poorer overall survival rate. Furthermore, multivariate analysis indicated that *CDK2AP1* expression was an independent prognostic factor in LIHC patients. The risk assessment model we established takes *CDK2AP1* expression as an important predictor, combined with other risk factors such as clinical stage, the predicted results are similar to the actual ones. As a prognostic biomarker, the application of *CDK2AP1* requires more clinical validation.

Immunotherapy plays an important role in the treatment of HCC (Ruf et al., 2021; Sangro et al., 2021). Our study used the TIMER database to reveal the link between *CDK2AP1* expression and immune infiltration in HCC. In order to explore the therapeutic potential of *CDK2AP1* in HCC immunotherapy, correlation analysis was conducted, which found that the correlation between *CDK2AP1* and neoantigens, MSI, TMB is weak. Subsequent correlation analysis of *CDK2AP1* and immune cell status showed that the differential expression of *CDK2AP1* may be related to activated B cell, activated CD4 T cell, activated dendritic cell and central memory CD8 T cell and other related immune pathways. According to *CDK2AP1* and checkpoint genes correlation analysis, *CDK2AP1* differential expression is associated with most checkpoint genes, but not with ADORA2A, BTNL2, IDO2, TNFRSF9 and TNFSF14. These results may provide new perspectives for HCC immunotherapy. Furthermore, we noticed that the expression of *CDK2AP1* was closely related to multiple tumor immune pathway as well as KICH, COAD and UVM checkpoint genes. We found that highly expressed *CDK2AP1* in HCC patients can trigger immune responses. Studies have proven that *CDK2AP1* plays a critical role in the regulation and recruitment of immune-infiltrating cells in the HCC microenvironment. All of these findings support the notion that *CDK2AP1* may be important in cancer immunotherapy.

Co-expression analysis showed that genes positively or negatively correlated with *CDK2AP1* were also associated with patient outcomes. KEGG and GO analysis further revealed changes in the level of related cellular pathways, and some immune-related pathways also appeared in them, which is consistent with the previous findings of *CDK2AP1* affecting immune cell infiltration. The above results suggest that *CDK2AP1* may regulate the immune function in tumors and then affect tumor progression.

Furthermore, proliferation and metastasis assays indicated a critical role of *CDK2AP1* in promoting tumor progression. The mice of control group had a poorer overall survival rate compared to *CDK2AP1* knockdown group, suggesting that *CDK2AP1* is critical for tumors. Intervention of *CDK2AP1* may have obvious benefits on the prognosis of liver cancer patients.

In the future, we can try to measure the expression of *CDK2AP1* in HCC surgical specimens to reflect the degree of malignancy of HCC patients, and even evaluate the local immune status of tumor tissues, so as to predict the efficacy of immunotherapy and the prognosis of patients. Although this study has given us some new insights into the treatment of liver cancer, it still faces some limitations. The specific impact mechanism between *CDK2AP1* and tumor development is still unclear, and further studies are needed to verify it.

## Conclusion

In summary, our study found that *CDK2AP1* expression level in HCC patient tissues was significantly higher than that in normal tissues. *CDK2AP1* is also considered to be an independent risk factor for HCC. Patients with high *CDK2AP1* expression have a poor prognosis. The nomogram model can effectively predict patient survival in clinical practice. The role of *CDK2AP1* in immunity remains to be further studied, and new targeted drugs may be developed. *In vitro* and *in vivo* experiments further validated the role of *CDK2AP1* in tumor growth and metastasis. Therefore, this study broadens our new understanding of hepatocarcinogenesis and suggests that *CDK2AP1* may serve as an effective target for HCC therapy.

## Data Availability

The datasets presented in this study can be found in online repositories. The names of the repository/repositories and accession number(s) can be found in the article/Supplementary Material.

## Abbreviations

BP, biological process; CC, cellular component; GO, gene ontology; KEGG, Kyoto encyclopedia of genes and genomes; MF, molecular functions; MSI, microsatellite instability; OS, overall survival; ROC, receiver operating characteristic; TMB, tumor mutation burden.
