# Supplementary material for: CDK2AP1 influences immune infiltrates and serves as a prognostic indicator for hepatocellular carcinoma
Source: Front Genet. 2022 Aug 29;13:937310. doi: 10.3389/fgene.2022.937310 (PMC9465009; doi:10.3389/fgene.2022.937310)
Supplement: Supplementary file 1 [file DataSheet2.PDF]

Figure S2

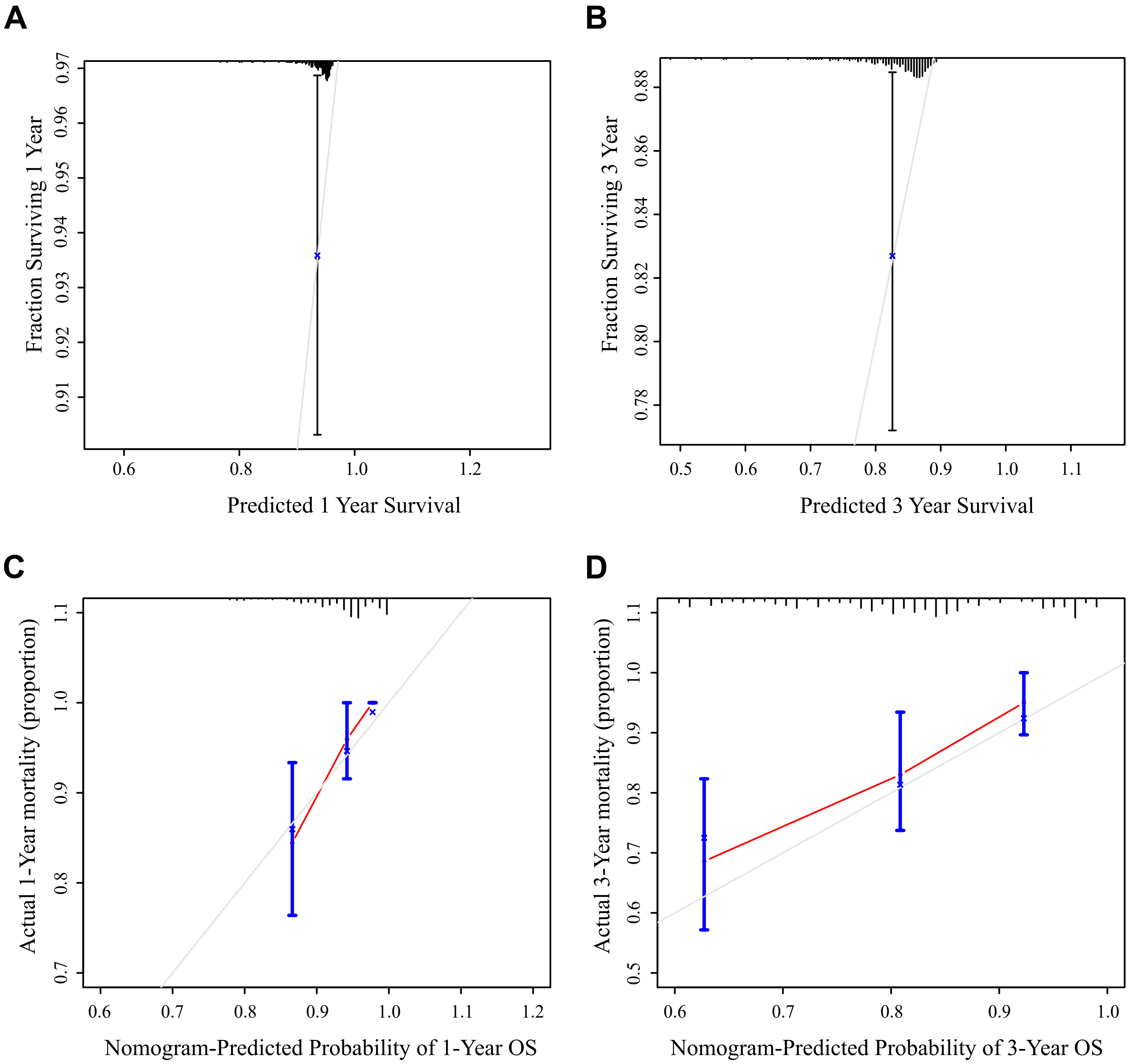

Supplementary Figure 2. Discrimination and calibration curves of the validation cohort. (A,B) The discrimination ability for 1-year (A) and 3-year (B) Overall Survival nanogram. (C,D) Calibration curves for 1-year (C) and 3-year (D) Overall Survival nanogram.
