# Supplementary material for: CDK2AP1 influences immune infiltrates and serves as a prognostic indicator for hepatocellular carcinoma
Source: Front Genet. 2022 Aug 29;13:937310. doi: 10.3389/fgene.2022.937310 (PMC9465009; doi:10.3389/fgene.2022.937310)
Supplement: Supplementary file 4 [file DataSheet1.PDF]

Figure S1

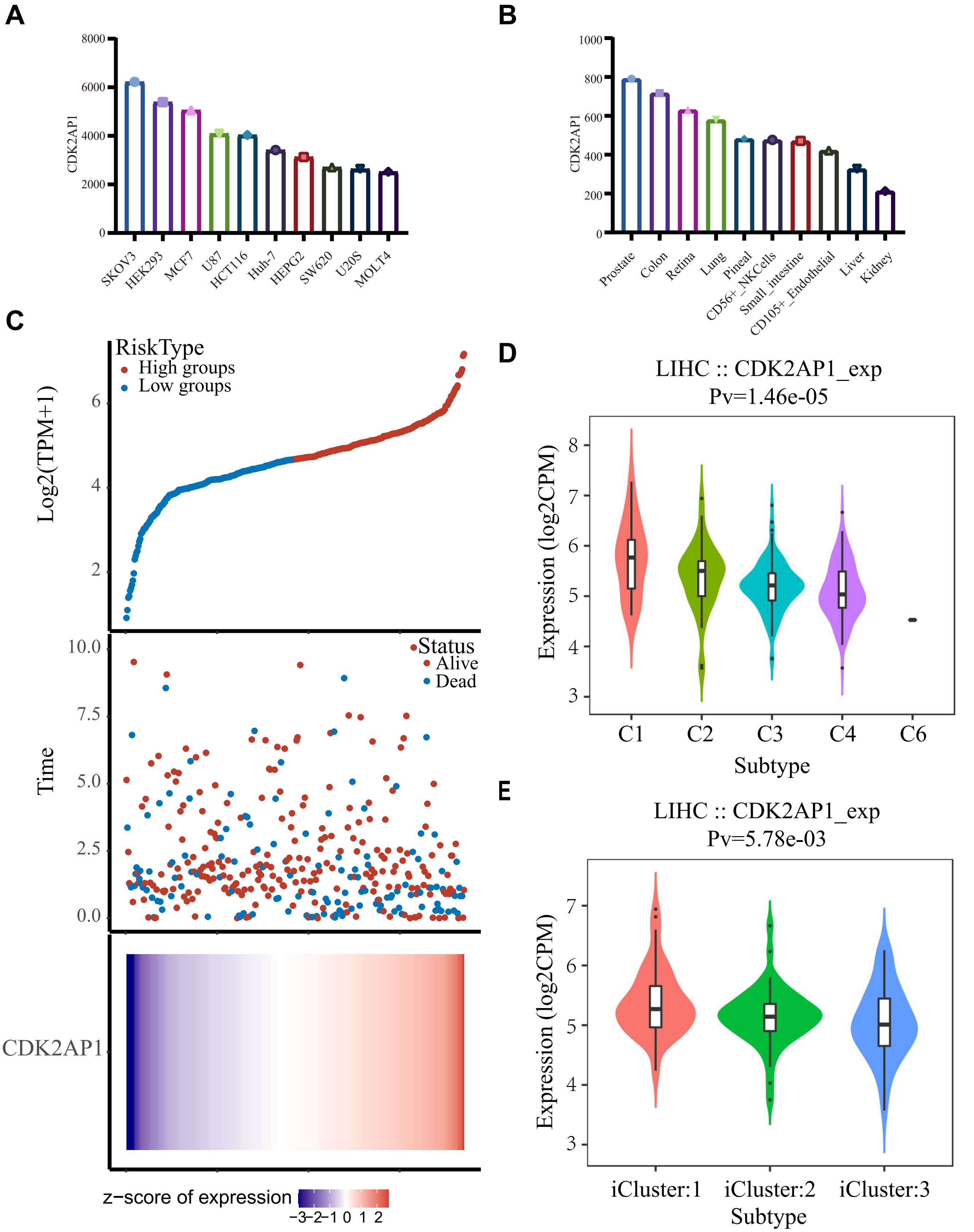

Supplementary Figure 1. Expression of CDK2AP1 in different cell lines, immune subtypes and molecular subtypes, and its effect on survival status. (A,B) The expression of CDK2AP1 in different cancer cell lines (A) and normal tissues (B). (C) CDK2AP1 expression distribution and survival status. (D) The relationship between CDK2AP1 expression and HCC immune subtypes. C1 (wound healing), C2 (IFN-gamma dominant), C3 (inflammatory), C4 (lymphocyte depleted), C5 (immunologically quiet) and C6 (TGF-b dominant). (E) The relationship between CDK2AP1 expression and HCC molecular subtypes.
